# In‐house quality check of external beam plans using 3D treatment planning systems — a DVH comparison

**DOI:** 10.1120/jacmp.v17i3.6020

**Published:** 2016-05-08

**Authors:** Ayyalasomayajula Anil Kumar, Roopa Rani Akula, Komanduri Ayyangar, Reddy P. Krishna, Srinivas Vuppu, P. V. Lakshmi Narayana, A. Durga Prasada Rao

**Affiliations:** ^1^ Swami Jnanananda Laboratories for Nuclear Research, Department of Nuclear Physics Andhra University Visakhapatnam India; ^2^ MNJ Institute of Oncology & Regional Cancer Center Hyderabad India; ^3^ International Cancer Center, MGMMT Bhimavaram India

**Keywords:** TPS, DVH, cobalt‐60, Eclipse, ROPS

## Abstract

This paper presents a new approach towards the quality assurance of external beam plans using in‐house‐developed DICOM import and export software in a clinical setup. The new approach is different from what is currently used in most clinics, viz., only MU and point dose are verified. The DICOM‐RT software generates ASCII files to import/export structure sets, treatment beam data, and dose‐volume histograms (DVH) from one treatment planning system (TPS) to the other. An efficient and reliable 3D planning system, ROPS, was used for verifying the accuracy of treatment plans and treatment plan parameters. With the use of this new approach, treatment plans planned using Varian Eclipse planning system were exported to ROPS planning system. Important treatment and dosimetrical data, such as the beam setup accuracy, target dose coverage, and dose to critical structures, were also quantitatively verified using DVH comparisons. Two external beam plans with diverse photon energies were selected to test the new approach. The satisfactory results show that the new approach is feasible, easy to use, and can be used as an adjunct test for patient treatment quality check.

PACS number(s): 87.55.dk, 87.55.D‐, 87.55.Qr

## I. INTRODUCTION

The importance of accurate treatment planning, delivery, and dose verification has been stressed in a recent editorial by Malicki^(1)^ where IMRT plans with 10% variations among various institutions were cited. It is now well accepted by radiation therapy community that, without proper QA and independent checks, errors propagate and perhaps result in patient injury. Currently, the verification of treatment planning involves either MU or point dose calculation or planar dose measurement based on QA phantom geometry. There have been several studies of comparing different planning systems reported in the literature. Ebert et al.^(2)^ reported DVH comparison of different planning systems from multiple treatment centers using different dose algorithms for DVH calculations. Their results showed significant variations in the minimum dose to the target. In another study Cheng et al.^(3)^ analyzed breast treatments using six different planning systems, and found variation of within ±5% between two planning systems in the dose given to the breast. Petric et al.^(4)^ reported the results of comparison of BrainSCAN planning system (Brainlab AG, Feldkirchen, Germany) with Eclipse (Varian Medical Systems, Palo Alto, CA) and discovered that minor differences exist between the planning systems. Tan et al^(5)^ described the comparison of six treatment planning systems for entrance and exit doses and concluded that there is 2%–3% disagreement among the systems. These studies focused on comparing planning systems purely to differentiate dose calculation accuracy. There are many publications describing QA guidelines developed by various agencies.^6^, ^7^, ^8^, ^9^, ^10^, ^11^, ^12^, ^13^, ^14^

The goal of our current project is to use a secondary planning system as a QA tool for independently checking treatment planning. With the availability of DICOM‐RT export and import and the access of low‐cost planning system such as ROPS (as supplied by Tirumala Jyothi Computer Systems, Secunderaba, India, and described at https://sites.google.com/site/tjcsrops), this checking becomes now not only feasible but also more comprehensive. The new QA approach involves checking each step of planning, such as visually checking the contours and the registration, checking all treatment machine and energy selections, field sizes and field weights, SSDs, and the wedges, checking dose prescription, monitor units (MU) calculation, and DVHs. There are major differences between the method presented in this presentation and the traditional QA method. In the proposed method, patient geometry is included as the basis of independent check. But the traditional QA is based on QA phantom for either point‐dose calculation or planar dose measurement. Another difference is that the target coverage and dose to the critical tissues are verified in the proposed method, but not in the methods currently used in the clinic.

Traditionally the QA methods have focused on comparing MUs and dose measurements in phantoms, and also comparing planar dose calculations with measurements and evaluation of gamma factors. The method reported in the current investigation is different from traditional methods in the sense that we compare the total plan using DVHs calculated from two different planning systems. The criterion used for acceptability of the QA is that the CTV DVHs have to agree to within 5%.

All the plans in the current study were actually done using the Eclipse planning system to treat patients. Subsequently as a part of the QA study these plans were repeated using the ROPS planning system and the resulting DVH curves were compared with those from Eclipse planning system. While the Eclipse planning system is more versatile and uses AAA algorithm, the ROPS planning system is a 3D planning system based on Clarkson scatter integration algorithm. The contours from Eclipse planning system were exported via DICOM ASCII files. The ROPS planning system has standalone programs to read the DICOM ASCII files and import the data into the planning system. Except for the coordinate system transformation, the data were imported without any alterations. The same DICOM images were used in both the systems.

## II. MATERIALS AND METHODS

After completing the planning on the Eclipse system, the plan data were exported in the DICOM format for planning verification. This data consisted of essentially three ASCII files: structure contours (RS), plan and beam data (RP), and dose data (RD) including dose‐volume histograms (DVH). The exported dose data were generated from the default dose grids on which the cumulated DVH calculation was based. The isocenter position and calculation point position were determined using Eclipse system coordinates and contained in beam data files.

### A. ROPS

ROPS TPS was commissioned for the cobalt‐60 machine using BJR25 data and for the Varian DHX linac using measured commissioning data for external beams of energies 6X and 16X. Dose rate data, field output data, and wedge factors were specific to the individual machine used. The comprehensive tests were performed to verify the correction and accuracy of data collection and machine modeling according to the AAPM report TG‐53. In addition, ROPS has been tested using certain measurements and Monte Carlo calculations for cobalt‐60 fields.^(15)^ The details of ROPS specifications can be found from the website at https://sites.google.com/site/tjcsrops.

Between the two planning systems there were many differences, which caused difficulty in using the DICOM files exported from Eclipse system. While the Eclipse system uses 512×512 matrix size for every slice of CT images, the ROPS system scales it down to a 256×256 matrix. While both systems define the x‐ and y‐axes in the plane of the axial images, the y‐axis in the Eclipse system follows the supine patient's anterior direction. In view of this, the Y coordinates have to be reversed before registering. Because of the different image resolution and different coordinate system, the exported contours which were displayed in Eclipse coordinates did not exactly register with the CT images. However, the exported DICOM files had all the required tags to properly register the contours. Another difference is that in the ROPS the image Z coordinates must be arranged from head to foot. In view of these differences, a coordinate transformation had to be used. In view of limitations on contour data size, the body contours in ROPS had to be filtered. The contour registration was actually viewed on each slice to verify the body contours matching each slice. In addition, the contour registration was double‐checked using DICOMan software (http://radonc.uams.edu/research/DICOMan and https://sites.google.com/site/dicomantx/) developed by Dr. Yulong Yan, UT Southwestern Medical Center, Dallas, Texas, USA.

After the contour match was verified, coordinate transformation was applied to the isocenter coordinates. Beams in ROPS were defined by copying the same treatment machine, energy, dimensions, and angles from the DICOM data. If an irregular shape was defined either by using a block or an MLC, this shape was imported into ROPS as irregular shape by copying block edges or MLC leaf positions. Dose was calculated by defining a grid using the limits of the body contour dimensions. A grid spacing of 5 mm was used to calculate dose to the entire matrix, while the Eclipse planning system used a spacing of 2.5 mm to calculate the dose. ROPS uses depth‐dose calculation for each point by ray‐tracing of CT pixels. After the dose calculations, the doses from all the beams were summed using the same beam weights as used by Eclipse. The dose‐volume histograms (DVH) were then evaluated with the same dose grids for both target and organs at risk (OAR). Calculated DVH data was compared to imported DVH data to check the target dose coverage and dose to OAR. Several clinical plans have been tested using this approach and in all cases the agreement criteria have been met. Two clinical case examples are presented in this paper.

## III. RESULTS

The first case was a four‐field plan using cobalt‐60 beams, and the second case a pelvic plan using 6 MV and 16 MV beams. Both the patients received a prescribed dose of 50 Gy in 25 fractions. The first case is for a cervix cancer treatment using four cobalt beams. Table 1 shows SSD comparison between the two systems for the first clinical case. It can be seen that the differences in SSDs are not very significant. The SSDs in ROPS were determined by ray‐tracing of CT pixels.

**Table 1:** SSD comparison.

*Beam*	*ECLIPSE (mm)*	*ROPS (mm)*	*Diff. (mm)*
AP	687	687	0
PA	722	722	0
RL	639	641	+2
LL	642	639	−3

Table 2 shows the treatment time comparison between the two systems. There seems to be a systematic difference in the treatment time computation. While this difference is significant, increasing the treatment time in ROPS would increase the dose per fraction and will not match the Eclipse plan. The Eclipse‐implemented DICOM‐RT does not have the information about dose rate or timer error and hence this difference in treatment time could not be resolved.

**Figure 1:**
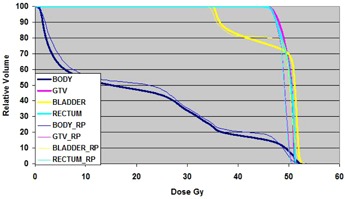
DVH Comparison for case 1. Thick lines are from Eclipse plan and thin lines are from ROPS.

Figure 1 shows the DVH comparison for this case. The Eclipse plan DVHs are shown as thick lines and the ROPS plan DVHs as thin lines. The GTV DVH is in good agreement within 0.5 Gy. The DVH for rectum for ROPS is 2 Gy less than Eclipse. There are no other significant differences.

Table 3 shows comparison of volume of the clinical structures. In view of the filtering of contours due to 256×256 matrix conversion, the agreement of structure volumes within 5% is considered reasonable. However, in some regions the agreement was as high as 10%.

Table 4 shows DVH parameters D95, V95 and V100 for the GTV. The D95 and D100 parameters are the dose received by 95% and 100%, respectively, of the volume. The V95 is the percent volume receiving 95% of prescription dose. It can be seen from the Table that the D95 and D100 values are in agreement between both the planning systems. There is a 5% discrepancy in the V95 values. This is because of the maximum dose in the ROPS plan was lower than in the Eclipse plan.

**Table 2:** Treatment time comparison.

*Beam*	*Eclipse (min)*	*ROPS (min)*	*Diff. (min)*
AP	0.84	0.79	0.05
PA	0.86	0.80	0.06
RL	0.82	0.73	0.09
LL	0.82	0.74	0.08

**Table 3:** Structure volume comparison.

	*Eclipse (ml)*	*ROPS (ml)*	*ROPS/Eclipse Ratio*
Body	14825	14198	0.96
Gtv	98	106	1.08
Bladder	346	362	1.04
Rectum	58	65	1.11

The second case is for a bladder cancer pelvic treatment using four linac beams. Opposing 6 MV AP/PA beams were used along with 16 MV lateral beams. Table 5 shows SSD comparison between the two systems for this clinical case. It can be seen that the differences in SSDs are not very significant except for the third beam. While this discrepancy can be corrected by shifting the isocenter by 1–2 pixels, that would change the SSDs of other beams.

**Figure 2:**
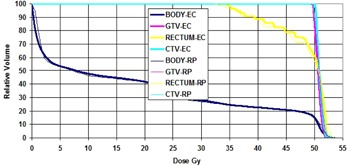
DVH Comparison for case 2. Thick lines are from Eclipse plan and thin lines are from ROPS.

Table 6 shows the MU comparison and can be seen to be in good agreement between the planning systems. Figure 2 shows the DVH comparison for this case. The Eclipse plan DVHs are shown as thick lines and the ROPS plan DVHs as thin lines. It can be seen that the agreement is good for all the structures.

7, 8 show the structure volume and DVH parameter comparisons, respectively. Good agreement can be seen between the two planning systems. The D100 shows a discrepancy of 2 Gy, which indicates dose nonuniformity in the target volume.

**Table 4:** DVH parameter comparison.

	*Eclipse*	*ROPS*	*Percent Diff.*
D95 (Gy)	47.4	47.0	−0.8%
V95 (%)	94%	90%	−4.0%
D100 (Gy)	45.17	45.0	0.4%

**Table 5:** SSD comparison.

*Beam*	*Eclipse (mm)*	*ROPS (mm)*	*Diff. (mm)*
1 AP	948	947	−1
2 PA	909	912	+2
3 LL	862	854	−8
4 RL	872	873	+1

**Table 6:** MU comparison.

*Beam*	*Eclipse*	*ROPS*	*MU Diff.*
1 AP	55	55	0
2 PA	70	68	−2
3 LL	50	49	−1
4 RL	48	47	−1

**Table 7:** Structure volume comparison.

	*Eclipse (ml)*	*ROPS (ml)*	*ROPS/Eclipse Ratio*
Body	10238	10304	1.01
GTV	129	131	1.01
Rectum	25	26	1.04
CTV	357	374	1.05

**Table 8:** DVH parameter comparison.

	*Eclipse*	*ROPS*	*Percent Diff.*
D95 (Gy)	49.81	50.0	0.4%
V95 (%)	100%	100%	0.0%
D100 (Gy)	49.5	47.5	−4.0%

### A. Establishing the need for QA of treatment planning systems

Initially while performing QA, many plans were checked for effective contour registration and its matching with the body skin.

**Figure 3:**
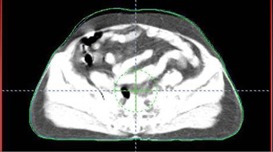
Agreement between Eclipse‐generated contour (green) and ROPS‐generated body contour (red).

Figure 3 shows Eclipse imported contour (green) and ROPS‐generated contour (red). This agreement proves that the contour registration process is validated.

**Figure 4:**
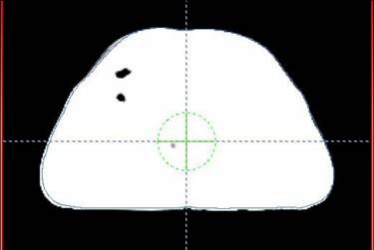
Contour mismatch when CT contrast is adjusted.

Figure 4 shows the contour matching with different contrast adjustment of the CT image. It can be seen that the same contour shown in Fig. 3 is now off by 1–2 pixels. In other words, skin edge detection is subject to a 1–2 pixel error. This discrepancy is usually due to the seed CT value used in tracking the outer contour.

**Figure 5:**
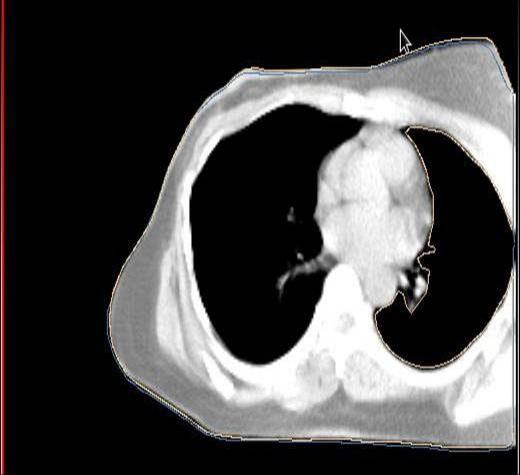
The body contour entered the lung due to image cutoff.

As a part of quality check, we have come across some situations where the CT image is cut off on purpose, such as a breast image shown in Fig. 5. In this breast treatment, the patient's position is shifted purposely to get the CT of the right breast. This cuts off a portion of the left of the patient scan. The body contour defined in Eclipse plan is as shown below, which excluded the left lung from the body contour. If the user does not intervene, and correct the contours, the body volume is miscalculated.

**Figure 6:**
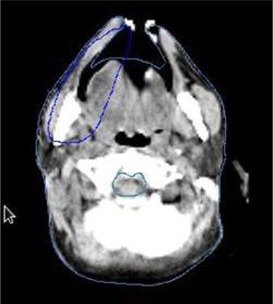
Body contour (green) entered the mouth and interferes with target contour (red).

Figure 6 depicts an image where a part of the target volume is outside the body contour. The target volume is in red and body contour in green. Depending on how the treatment planning system defines the body tissues, dose can be zero in the target volume outside the body contour. In such case, it becomes necessary to recheck and edit the body contours so as not to enter the oral cavity for efficient treatment delivery. The method of DICOM import and verifying the plan accuracy presented here serves this purpose.

## IV. DISCUSSION

Implementation of the new method requires the import of the DICOM‐RT structures (RS), plan beams data (RP), and the dose files (RD). The implementation of DICOM‐RT is especially difficult for plan export, since the user could define many plans, beams, and fiducial points, and may not use them all in the final plan. This poses many issues to the planning system manufacturer. One of these issues is the system may not know what to export. Unlike DICOM‐RT exporting, importing RS and RP would need to reformat the data based on the new planning system configuration. Hence, some planning systems have not supported the RP export. The ROPS planning system primarily depends on the imported and reformatted RS and RP data to generate a QA plan. The QA plan is completely identical to the plan used in conventional QA measurement, but it is developed from patient geometry rather than QA phantom. The RD file would be needed in comparing the DVHs. The final DVH comparison was made outside ROPS system manually and the comparison results were analyzed quantitatively to show the target dose coverage and dose to OARs which would be recorded as the treatment planning verification results.

This method is not limited to ROPS planning system. Many institutions already have more than one planning system. It is possible to adapt this method between the planning systems and develop an additional QA method than what they are currently using. Recently another software system, Mobius (Mobius Medical Systems, LP, Houston, TX), has already implemented DVH comparison^(16)^ as a QA tool.

A small isocenter position shift would cause significant variation of dose calculation. While it is best to import the beams for good accuracy, we were able to define the isocenter using the RP data import and define the beams using the usual beam parameters, such as field size, gantry and collimator angles, and wedge. If the RP import is not available, it is possible to define the isocenter and beams manually by visually examining the location of the isocenter and getting all the other parameters from treatment data. This may be the only way to use this method for checking a treatment plan with some planning systems that do not support the export of beam information.

It should be noted that the current investigation is limited to reporting the in‐house approach developed for the QA purpose. This is neither an endorsement of the planning system nor criticism of the QA software. It would take a major effort to make any planning system compliant with FDA regulations in order to accept data from different planning systems. What we emphasize in this study is that the discussed method would report the results of comparing dose to target and important organs, instead of MU or point‐dose calculation.

It should also be noted here that doing QA of a plan this way would reduce the risk for a plan to be undeliverable, but not eliminate the need to verify the plan on the machine. After all, the plan deliverability should be tested by loading all treatment parameters from the R&V system to the console computer and verified by clearing all errors in machine control interface.

## V. CONCLUSIONS

It has been demonstrated through the case studies shown above that there is no significant difference between the DVH curves generated from two different planning systems. With the use of the present new approach, The ROPS treatment planning system is capable of generating treatment plans equivalent to Eclipse. Hence, ROPS offers a low‐cost solution to QA check treatment plans. In addition, this method of independent DVH calculation and comparison is helpful in developing a QA procedure for treatment planning systems. This method can be implemented in institutions that have invested in multiple planning systems.

## ACKNOWLEDGMENTS

The authors wish to thank the Atomic Energy Regulatory Board for the award of a grant through which this work was conducted. The authors wish to thank Dr. Sicong Li, University of Nebraska Medical Center, Omaha, NE, for helpful suggestions. The authors also wish to express their sincere thanks to Dr. A.R. Reddy, M. Palani Kannu, and V.V. Reddy, physicists of MGMMT cancer center, for their review and assistance with Eclipse treatment planning.

## COPYRIGHT

This work is licensed under a Creative Commons Attribution 4.0 International License.
